# Fear and Stigma: The Epidemic within the SARS Outbreak

**DOI:** 10.3201/eid1002.030750

**Published:** 2004-02

**Authors:** Bobbie Person, Francisco Sy, Kelly Holton, Barbara Govert, Arthur Liang, the NCID, SARS Community Outreach Team (listed in alphabetical order), Brenda Garza, Deborah Gould, Meredith Hickson, Marian McDonald, Cecilia Meijer, Julia Smith, Liza Veto, Walter Williams, Laura Zauderer

**Affiliations:** * Centers for Disease Control and Prevention, Atlanta, Georgia, USA; † Northrop Grumman Mission Systems, Atlanta, Georgia, USA

**Keywords:** Asian-Americans, community outreach, discrimination, fear, health education, hotlines, outbreaks, risk behaviors, SARS, stigmatization

## Abstract

Because of their evolving nature and inherent scientific uncertainties, outbreaks of emerging infectious diseases can be associated with considerable fear in the general public or in specific communities, especially when illness and deaths are substantial. Mitigating fear and discrimination directed toward persons infected with, and affected by, infectious disease can be important in controlling transmission. Persons who are feared and stigmatized may delay seeking care and remain in the community undetected. This article outlines efforts to rapidly assess, monitor, and address fears associated with the 2003 severe acute respiratory syndrome (SARS) epidemic in the United States. Although fear, stigmatization, and discrimination were not widespread in the general public, Asian-American communities were particularly affected.

 Public health strategies that deal with rapidly evolving disease outbreaks of new and emerging infectious diseases require a delicate balance between protecting the public’s health and initiating exclusionary practices and treatments that can lead to fear and stigmatization of, and discrimination against, specific populations. The outbreak of severe acute respiratory syndrome (SARS) illustrates these difficulties. SARS spontaneously appeared in the southern province of Guangdong, People’s Republic of China, in November 2002 (*1*,*2*). By July 2003, the epidemic, had spread to more than 30 countries with 8,427 cumulative probable cases and 916 deaths and was identified as a global threat to health (*1*). In the United States, 418 cases were reported with 74 classified as probable SARS; no deaths occurred (*1*). As with many disease outbreaks, scientific information and data related to the disease changed almost hourly, as public health scientists and practitioners responded to the worldwide outbreak, which was coupled with widespread fear (*3*,*4*).

## SARS-related Fear, Stigmatization, and Discrimination

While persons, agencies, and governments sought to identify modes of transmission, strategies for disease containment, and treatment for SARS, fear spread unchecked throughout the global community. Fear of SARS arose from the underlying anxiety about a disease with an unknown cause and possible fatal outcome (*5*). Stigmatization of potential SARS patients emerged early in the outbreak, as global media reported dramatic stories from Asia in print media, television, and the Internet. Headlines from the English-language press heightened the fear. “Concern is mounting over the continuing spread of the deadly SARS virus. Some experts say it could have a similar impact to the 1918 flu epidemic that killed 50 million—or the current world HIV crisis,” wrote the British Broadcasting Corporation from London, England (*6*). “China has threatened to execute or jail for life anyone who deliberately spreads the killer SARS virus,” stated the Cable News Network from Beijing, China (*7*).

 Studies have shown that during serious disease outbreaks, when the general public requires immediate information, a subgroup of the population that is at potentially greater risk of experiencing fear, stigmatization, and discrimination will need special attention from public health professionals (*8*–*10*). The recent SARS outbreak was a classic example of such an outbreak.

Fear is further fueled when infection control techniques and restrictive practices such as quarantine and isolation are employed to protect the public’s health (*11*,*12*). While exclusionary practices based upon the best available scientific evidence may be scientifically and ethically sound for one population, those same practices may not be sound for all populations (*5*,*11*). During the SARS outbreak, some persons became fearful or suspicious of all people who looked Asian, regardless of their nationality or actual risk factors for SARS, and expected them to be quarantined. Some Americans did not understand that quarantine and isolation practices appropriate for SARS-affected areas in Asia, where community transmission was a concern, were practices that were not appropriate in the United States where the disease was not community acquired. For example, some persons, who had recently traveled to areas where SARS was spreading, isolated themselves, even though they had no symptoms and had not been exposed to someone with SARS.

## Mitigating Fear, Stigmatization, and Discrimination through Strategic Community Outreach

Fear of being socially marginalized and stigmatized as a result of a disease outbreak may cause people to deny early clinical symptoms and may contribute to their failure to seek timely medical care (*5*). Such fear can ultimately increase stigmatization when cases are identified at a later date (*11*). Stigmatization associated with discrimination often has social and economic ramifications that intensify internalized stigmatization and feelings of fear (*13*).

Containing fear, which is integral to the public health management of a new and emerging disease such as SARS, is best accomplished by a behavioral strategy that addresses the needs of a segment of the population at risk of becoming stigmatized and discriminated against. This strategy works best as a complement to a larger public health education and communication campaign. Typically during outbreaks, initial risk communication is targeted to front-line public health professionals through vehicles such as the Morbidity and Mortality Weekly Report. Initial communication provides information on case definitions and laboratory-testing strategies, as well as interim guidelines for infection control and other critical issues. Communication strategies for the general public most frequently involve television sound bites, press conferences with dignitaries and health officials, and targeted release of information to mass media outlets such as newspapers and Internet sites (*14*). Although these risk communication activities are critical for keeping the general public informed during an outbreak, they can fail to meet the personal needs of the affected population and the general public.

## Methods

During the first week of April 2003, the National Center for Infectious Diseases (NCID) at the Centers for Disease Control and Prevention (CDC) formed a 14-member, multidisciplinary NCID/SARS Community Outreach Team as part of its emergency response to the global SARS outbreak. While other NCID/CDC response teams dealt with laboratory investigations, surveillance, communication, and clinical infection control practices, the Community Outreach Team worked to implement rapid public health strategies to document, monitor, and assist in ameliorating specific problems associated with fear, stigmatization, and discrimination attributed to the SARS outbreak in the United States.

In creating a rapid public health intervention to mitigate behaviors and practices associated with SARS-related fear, the team recognized the need to address the experiences of persons at greatest risk for experiencing SARS-related fear, stigma, and discrimination. The team monitored stigmatizing ideas and behaviors in the general population and the media, particularly toward Asian Americans, who were disproportionately reporting fear, stigmatization, and discrimination compared to the general public. The team began working with Asian-American communities to develop a culturally tailored intervention that 1) promoted community understanding of the facts related to the transmission and prevention of SARS; 2) contributed to the strengthening of community resiliency and capacity to mitigate fear, stigmatization, and discrimination; and 3) encouraged appropriate health-seeking behaviors for those who may have been exposed to SARS and were experiencing early symptoms. The team also worked to dispel myths; keep the general public better informed; prevent discrimination against SARS-affected communities; and provide guidance for institutions, agencies, and organizations hosting international visitors from SARS-affected countries.

### Rapid Situational Assessment

During the first 3 weeks of April 2003, the NCID/SARS Community Outreach Team conducted a rapid situational analysis to determine the impact of SARS-related fear, stigmatization, and discrimination within the Asian-American community in the United States. The team carried out the following activities: 1) facilitated group discussions with key opinion leaders within the Asian community in the United States; 2) collected and monitored the CDC Public Response Service data; 3) collected and monitored Asian-language newspapers, Internet sites, and other information sources; 4) reviewed polling data and other communication information; 5) conducted community visits, panel discussions, and media interviews; 6) solicited information from state and regional minority health liaisons nationwide; 7) developed ongoing relationships with the Asian-American communities; particularly in major metropolitan areas throughout the United States; and 8) determined new data-gathering strategies as needed.

### Group Discussions

The team conducted group interviews through teleconferences with national, state, and local influential leaders in the Asian-American community throughout the United States. The team also conducted group interviews with Chambers of Commerce and trade association members, school officials and representatives, state public health department staff, academicians at universities, mental health professionals, and others. The 11 teleconferences the team conducted reached more than 70 persons who represented more than 50 agencies, organizations, and communities. The goals of the group interviews were the following: 1) determine the impact of SARS-related fear on the Asian community; 2) document examples of fear, stigmatization, and discrimination; 3) determine strategies for identifying and reaching “hidden populations”; 4) develop partnerships with leaders and community members of the affected populations; 5) determine the needs of affected populations; and 6) respond appropriately to those needs through a targeted intervention with activities and Asian-language materials.

Five major recommendations were derived from the facilitated group discussions with key informants: 1) develop simple, tailored SARS prevention messages; 2) develop SARS information materials in various Asian languages; 3) disseminate SARS information through multiple and culturally appropriate channels, including (but not limited to) community visits, town hall meetings, and health education and communication channels to complement mass media messages; 4) establish partnerships with local Asian-American community–based organizations to educate the community; and 5) ensure that CDC would continue to provide leadership and coordination in preventing and controlling SARS. The relationships developed during these group discussions allowed team members to monitor and document ongoing stigmatizing situations related to the disease outbreak in real time and to deal more effectively with intentional and unintentional discrimination.

### CDC Public Response Service

CDC operates the Public Response Service (CDC PRS) under contract with the American Social Health Association. This contract provides hotline service to the general public requesting information via telephone and email about bioterrorism and other disease emergencies, including SARS. The NCID/SARS Community Outreach Team worked with the CDC PRS to track a daily sample of incoming SARS-related calls, specifically noting questions associated with fear, stigmatization, and discrimination directed toward the Asian-American community. This system allowed the team to help determine specific answers to frequently asked questions for hotline staff and to develop simple, prerecorded Asian-language messages. Passive data collection of SARS fear-related concerns began on April 29, 2003. During May 2003, 7,327 SARS-related calls were received; 4,013 (54.7%) of these calls were passively sampled. Of these sampled calls, an average of 10% of callers expressed concerns related to fear, stigmatization, and discrimination. A caller could express more than one concern. Major concerns included the following: fear of buying Asian merchandise (187 calls); working with Asians (83 calls); living near Asians (45 calls); going to school with Asians (41 calls); and more generic issus such as being on a cruise ship or airplane (77 calls); and church, school, or workplace issues (65 calls). Most SARS calls related to transmission, symptoms, and treatment of disease and travel advisories.

### Asian-Language Information Sources

One critical component of the team’s activities was determining where members of the Asian-American community were getting SARS-related information. Team members monitored English-language and Asian-language electronic, print, and television media coverage and informal chat rooms in the United States and other countries to stay abreast of changing information about the nature of the SARS outbreak that could influence fear, stigmatization, and discrimination. The assessment showed that many people within the Asian-American community were getting information from Asian-language newspapers, television, and Internet sites directly from China, Hong Kong, Taiwan, and other Asian areas—usually hours ahead of information providers in the United States. The information provided by these Asian-language sources was often inconsistent with newspaper, television, and Internet coverage in the United States, thus creating fear and suspicion that the United States government might not be telling the truth about the outbreak in this country. Independent content-analysis research conducted by InterTrend Communications (San Francisco, CA) compared four of the most popular Chinese language newspapers in the United States with two popular national mainstream English-language newspapers from March 21 to April 3, 2003 (*15*). InterTrend data showed that 1) Chinese-language newspapers were more likely to highlight SARS news related to the Chinese community in the United States or from China more prominently than mainstream English-language newspapers; and 2) Chinese-language newspapers were more likely to have articles on SARS, including featured in-depth articles, than mainstream English-language U.S. national newspapers (*15*). These findings supported the team’s initial assessment (based on an informal convenience sample of Asian-language papers).

General email inquiries sent to the CDC communications center and information from public health professionals, health providers, and community members led the team to SARS-related Internet sites that contained rumors and inaccurate information, which added to general misunderstanding, confusion, and fear. Even legitimate public health Internet sites from different parts of the world provided disparate information as the outbreak unfolded, furthering uncertainty and fear in the United States. The team also monitored Internet sites that supported community fears as they promoted home remedies, medicinal cures, and inappropriate and unnecessary protective equipment. Monitoring the information sources of the affected population was a critical activity, allowing the team to separate fact from fiction with accuracy and timeliness and address salient issues and concerns during community visits.

## Results

### Rapid Situational Response

Based on its rapid situational assessment, the team was able to develop interventions to assist in mitigating fear, stigmatization, and discrimination. Team members carried out the following activities: 1) advised other SARS emergency response teams on how to minimize the risk of stigmatizing groups in their own communications by focusing messages on the virus and the relevant behavioral risk factors; 2) assisted with developing culturally tailored health education materials; and 3) conducted community visits, panel discussions, and media interviews to positively influence negative behaviors occurring in communities. These visits and other contacts with the Asian-American community allowed CDC to develop ongoing relationships and helped the team determine new data-gathering strategies.

### Targeted Health Education Materials

During a disease outbreak, information changes rapidly as scientific evidence is collected and analyzed. Vital components of the team’s activities were prioritizing and translating existing information and guidance documents and developing health education materials to address the specific needs of the Asian-American community. An in-house translation service did not exist, and the rapidly evolving scientific evidence challenged the turnaround time for developing, translating, and disseminating information. The team worked to identify priority documents for translation and to ensure Asian-language translation for Web and print products tailored to the Asian-American community. To ensure accurate translations, CDC contracted with professional translation services and had all documents back-translated. Web-based information on SARS included documents in traditional Chinese, simplified Chinese, Korean, Vietnamese, and Japanese, as well as French and Spanish. The team also created brief, recorded educational hotline messages in Chinese and Vietnamese. The main messages for people in the United States were the following: 1) the risk of SARS is low; 2) severe cases of SARS have been uncommon, and there have been no deaths in the United States; 3) methods for disease prevention in the general public are like those of other viral diseases; and 4) although no evidence of community spread currently exists, continued vigilance, aggressive case management, and infection control are needed.

### Community Field Visits

Team members conducted field visits to Asian communities in Boston; New York City; Oakland, California; San Francisco; Washington, D.C.; Edison, New Jersey; and Los Angeles to respond to the direct needs of the communities and gather information. The team met with community leaders, toured the communities, informally gathered further information, and gave community SARS presentations in seven cities, reaching approximately 500 persons. Through community visits, the team was able to 1) provide the latest in evidence-based information on SARS with Asian-language education materials; 2) dispel misconceptions, myths, and rumors; 3) act as a catalyst for bringing together a broad spectrum of organizations and persons in the community to create local networks to promote community resiliency; and 4) provide credibility and reassurance to those who felt vulnerable. Speakers also presented a public health model for mitigating fear, stigmatization, and discrimination that could be instituted by public health officials, clinicians, and community members. Through open discussion sessions and informal information gathering in the community, the team found that SARS-related stigmatization was occurring more frequently within the Asian community than from outsiders directed toward the Asian community. The team also found that those persons with SARS-like symptoms who used traditional herbal physicians and pharmacies were less likely to be referred to, or seek out, public health officials, suggesting that further research into strategies to reach this population is needed. Conducting community visits also showed that CDC was responding to the needs of the community at risk for SARS-related fear, stigmatization, and discrimination and was modeling positive behaviors to the public.

## Discussion

Other infectious disease epidemics have been associated with specific ethnic groups. Fear, stigmatization, and discrimination plagued Russian Jewish immigrants when the 1892 outbreaks of typhus fever and cholera in New York City were traced to Russian Jewish immigrants from Eastern Europe (*8*). In the spring of 1900, the Chinatown community in San Francisco was faced with extreme discrimination due to an outbreak of bubonic plague, the “black death,” attributed to rats transported on a ship from Hong Kong (*9*). In 1993 an outbreak of hantavirus infection in the Four Corners area (where the borders of four states—Arizona, New Mexico, Utah, and Colorado—meet) of the United States was initially referred to by reporters as a Navajo disease, which led to severe fear, stigmatization, and discrimination of Native Americans in the region (*10*). Previous scientific studies have shown that fear associated with stigmatization and discrimination has negatively affected public health efforts with chronic conditions and diseases such as mental illness, HIV/AIDS, tuberculosis, leprosy, and epilepsy (*16*–*20*). More recently, stigmatization associated with fear and the AIDS epidemic negatively influenced voluntary testing, counseling, and treatment of those infected with the disease (*21*). Health providers have also seen reluctance by recent refugees and immigrants to get tested and treated for tuberculosis because of possible social stigmatization (*22*). The potential of being labeled at-risk for having or transmitting a stigmatizing condition such as SARS creates fear and anxiety, and an entire population of people can be at risk for becoming stigmatized in society (*23*).

Protecting the health of the public while preventing stigmatization of segments of the population during a rapidly evolving disease outbreak is complex. The team’s experience during the recent SARS outbreak demanded anticipatory insight, perceptive planning, and a rapid response to a targeted audience with specific cultural perspectives and influences. It also required us to recognize the distinctive features of SARS in a medical, social, and cultural context. Weiss states, “Preventing fear and stigmatization depends on controlling or treating the target health problem, countering tendencies of those who stigmatize others, and supporting those who are stigmatized through emotional support and social policies” (*11*).

The data collected during the rapid situational assessment were critical in guiding activities of the team. Both the data and the data collection process assisted the team in establishing interpersonal relationships with community leaders, determining priority needs, identifying responsible intervention strategies, and developing effective communication channels. The team was able to better understand community perceptions and attitudes by identifying the communities’ trusted sources of information. When conducting community visits, the team was able to address discordant information, myths, and rumors; provide simple Asian-language messages and materials; and act as a catalyst to build community resiliency and prepare for the possibility of future emerging diseases. The team was also able to keep CDC/NCID leaders informed and to intervene when they identified discriminatory policies, practices, and actions that were inconsistent with evidence-based public health recommendations and guidelines.

Quelling fear-driven stigmatization and discrimination during the SARS outbreak required tailored intervention strategies carried out by the SARS Community Outreach Team. These activities complemented traditional risk communication for the general public. To be effective, behavioral intervention approaches, messages, and materials had to be salient for the affected population, in this case Asian-American communities within the United States. Further, these interventions aimed at promoting an accurate understanding of the epidemic both in the general population and within the affected community, that is, the dynamic nature of the outbreak and its cause, treatment options, and prevention strategies. Through interpersonal connections, the team members worked to promote reassurance and enhance community resiliency.

Public health professionals must understand the necessary balance needed to protect the public’s health with appropriate exclusionary practices, while at the same time preventing fear, stigmatization, and discrimination of specific segments of the population. As we prepare for the next new or reemerging disease outbreak, we should also be preparing to deal with the fear epidemic that will likely accompany it. By developing effective behavioral and health education strategies and providing timely attention to the special needs of affected populations, we can ensure that, no matter what the infectious disease, we can limit the associated epidemic of fear and stigmatization.
